# Diagnostics for Lassa fever virus: a genetically diverse pathogen found in low-resource settings

**DOI:** 10.1136/bmjgh-2018-001116

**Published:** 2019-02-07

**Authors:** Laura T Mazzola, Cassandra Kelly-Cirino

**Affiliations:** FIND, Emerging Threats Programme, Geneva, Switzerland

**Keywords:** Lassa fever virus, LASV, in-vitro diagnostics, outbreak

## Abstract

Lassa fever virus (LASV) causes acute viral haemorrhagic fever with symptoms similar to those seen with Ebola virus infections. LASV is endemic to West Africa and is transmitted through contact with excretions of infected *Mastomys natalensis* rodents and other rodent species. Due to a high fatality rate, lack of treatment options and difficulties with prevention and control, LASV is one of the high-priority pathogens included in the WHO R&D Blueprint. The WHO LASV vaccine strategy relies on availability of effective diagnostic tests. Current diagnostics for LASV include in-house and commercial (primarily research-only) laboratory-based serological and nucleic acid amplification tests. There are two commercially available (for research use only) rapid diagnostic tests (RDTs), and a number of multiplex panels for differential detection of LASV infection from other endemic diseases with similar symptoms have been evaluated. However, a number of diagnostic gaps remain. Lineage detection is a challenge due to the genomic diversity of LASV, as pan-lineage sensitivity for both molecular and immunological detection is necessary for surveillance and outbreak response. While pan-lineage ELISA and RDTs are commercially available (for research use only), validation and external quality assessment (EQA) is needed to confirm detection sensitivity for all known or relevant strains. Variable sensitivity of LASV PCR tests also highlights the need for improved validation and EQA. Given that LASV outbreaks typically occur in low-resource settings, more options for point-of-care testing would be valuable. These requirements should be taken into account in target product profiles for improved LASV diagnostics.

**Summary box**

- Diagnostics are key to effective prevention and control of Lassa fever virus (LASV), a WHO R&D Blueprint priority pathogen that causes acute viral haemorrhagic fever.
- Current diagnostics include laboratory-based serological and nucleic acid amplification tests as well as rapid diagnostic tests.
- Challenges to LASV diagnostics include commercial availability of clinically validated pan-lineage tests, few options for point-of-care testing, differentiation from other agents that cause similar symptoms and a need for improvements to test validation, regulation and external quality assessment; target product profiles for LASV diagnostics should be refined to take into account these needs.

## Introduction

Lassa fever virus (LASV) causes acute viral haemorrhagic fever (VHF) and is endemic to West Africa. Every year, approximately 100 000–300 000 people contract LASV, and 5000 people die from the infection.^1–3^ The recent 2018 outbreak in Nigeria saw 423 confirmed cases with a case fatality rate of 25%,^4 5^ and higher case fatality rates of up to 50%–70% have been reported.^6 7^ Because of its potential for zoonotic and human transmission as well as difficulties in treatment and prevention, LASV is one of the high-priority pathogens identified on the WHO R&D Blueprint.^1 2 8 9^ In June 2017, WHO finalised a LASV vaccine strategy,^10^ which relies on improved diagnostic tests as well as enhanced surveillance capacity in endemic countries. Here, we summarise existing LASV diagnostics and highlight remaining research and development needs.

### Epidemiology

**Figure 1:**
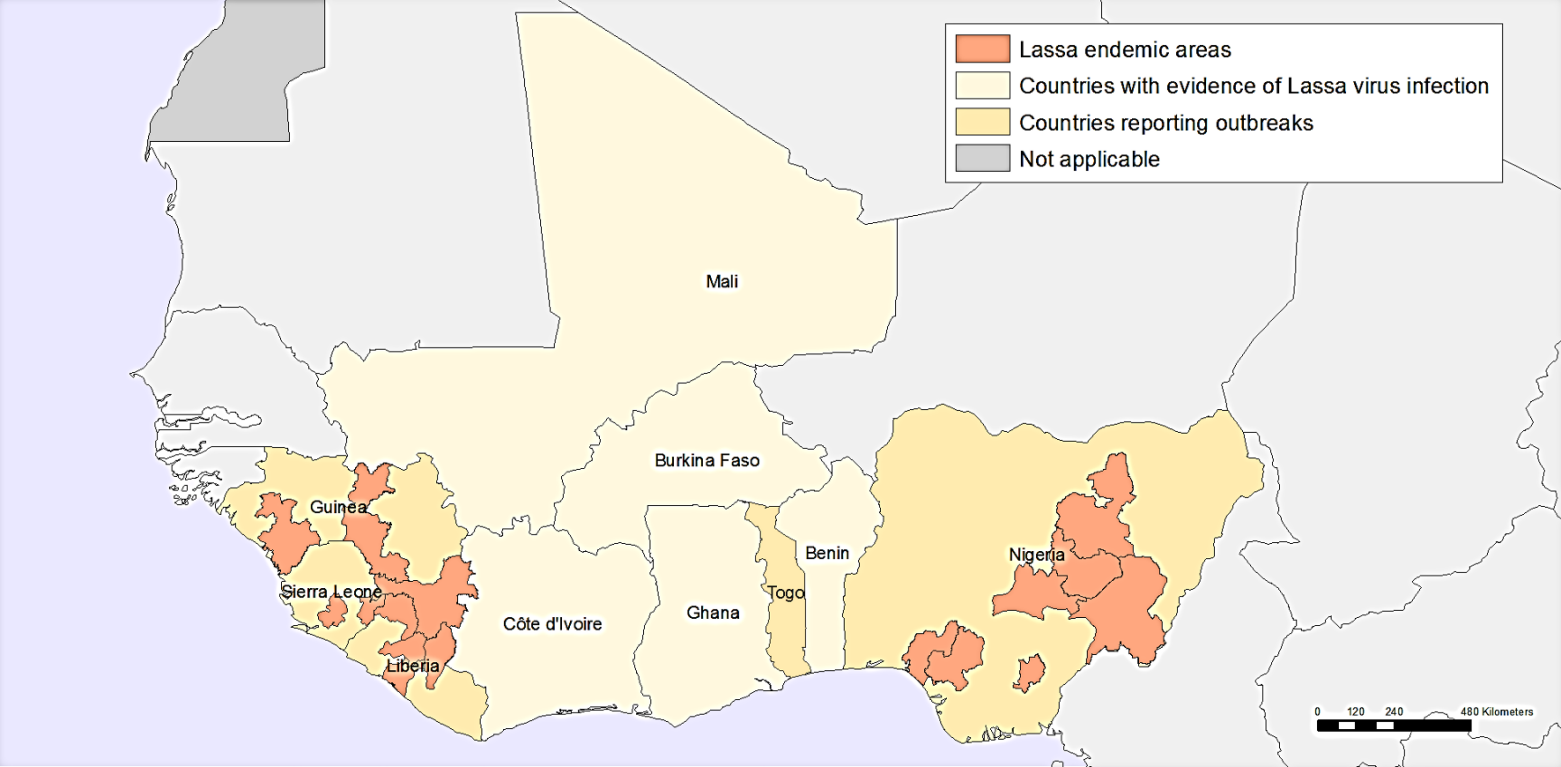
Geographic distribution of Lassa fever in West Africa. Adapted from Emergencies—Lassa fever, WHO, Geographic distribution of Lassa fever in west African affected countries, 1969–2018, Copyright 2018.

LASV is a single-stranded RNA virus of the *Arenaviridae* family. First identified in 1969 in Nigeria,^11^ Lassa fever is now endemic in West Africa including Nigeria, Sierra Leone, Guinea, Liberia, Benin, Ghana and Mali and has spread to neighbouring countries (figure 1).^12–15^ In some areas, 10%–16% of people admitted to hospitals every year have LASV.^1^ Cases have also been identified in Germany,^13 15 16^ the Netherlands,^17 18^ Sweden,^19^ the USA,^20–22^ the UK^23 24^ and Japan,^25^ largely imported after travel in West Africa.^17 26–28^ The long incubation period of LASV (~7–10 days) makes it one of the most commonly exported VHFs to countries outside its endemic range.

### Reservoir

*Mastomys natalensis* multimammate rodents are the most common rodent across the African continent, found predominantly in rural areas and human dwellings.^29–32^ These rodents show persistent LASV infection but are largely unaffected by the disease and shed the virus in their excrement.^33^ Seroprevalence has been reported to be as high as 60%–80% in *M. natalensis* populations.^29 34 35^ More recently, other rodent species including *Hylomyscus pamfi* and *Mastomys erythroleucus* have been shown to host LASV.^36 37^ Transmission to humans occurs primarily through contact with infected rodent urine or faeces; handling and consumption of infected rodents is also a pathway to infection.^32 38^ Airborne transmission may occur from aerosolised rodent excretions (dust) during cleaning activities.^1 2^ *M. natalensis* rodents readily colonise human areas where food is stored, contributing a significant risk for spillover, especially in communities with poor sanitation or crowded living conditions.^1 2^

Human-to-human transmission is less common, but LASV can be spread through direct contact with bodily secretions of persons infected with Lassa fever, presenting a higher risk for healthcare and humanitarian personnel,^39–42^ which increases with progression of disease and increasing viral load.^41 43^ There are suspected sexual transmission risks, as LASV can be detected in semen for 3 months past symptomatic infection.^32 44 45^

### Prevention and control

Prevention of Lassa fever relies on promoting good community hygiene to reduce the potential for human-rodent contact. Measures to discourage rodents include storing grain and other food in rodent-proof containers, good hand and food hygiene, disposing of garbage away from the home, maintaining clean households and trapping rodents or employing cats as a natural deterrent.^1 2 46^ Regular and sustainable environmental sanitation is also needed to reduce rodent activity. Although rodents are a food source for a high percentage of some communities, consumption should be discouraged.^47^

Healthcare settings should employ standard infection prevention and control precautions when caring for patients.^40 42 45 48–51^ Healthcare and laboratory workers should handle LASV specimens under maximum biosafety level 4 (BSL-4) biological containment conditions where possible.^52 53^ If BSL-4 precautions are not available, samples may be handled in a class II/III biosafety cabinet under BSL-2 precautions.^1 2^

Early detection is critical for LASV containment, and a strong surveillance system is necessary to support interventions in endemic or ‘hot spot’ areas for LASV and other VHF.^53 54^ WHO and partners support national authorities in affected countries for outbreak preparedness and emergency response once an outbreak has been detected,^55^ as shown in recent outbreaks in Nigeria.^56–60^

### Clinical indications and management

Early detection of LASV infection is difficult as the clinical course is highly variable, with symptoms ranging from 2 to 21 days postinfection. Lassa fever symptoms can mimic other endemic diseases such as malaria, typhoid fever and other VHFs.^1 2^ Although bleeding may help to discriminate VHF from other febrile illness, only 30% of patients with Lassa fever present with visible bleeding.^7 42 61 62^

Around 80% of people who become infected with LASV are asymptomatic or have mild symptoms of gradual fever, weakness and malaise, which often go unreported.^32 63^ After a few days, roughly 20% of infections progress to headache, sore throat, cough, muscle/joint pain, chest/abdominal pain, nausea, vomiting or diarrhoea. Indications of severe infection include facial swelling, fluid in the lung cavity, low blood pressure, petechiae and bruising, hepatitis and haemorrhaging of the conjunctival, gastrointestinal or mucosal tissue. Critically ill cases often present acute respiratory distress, shock, seizures, tremor, disorientation and coma. Death occurs within 14 days of onset for 15%–20% of severe cases. Young people and pregnant women are disproportionately impacted by LASV; the disease is especially serious in the third trimester, with maternal and fetal mortality reaching rates of 80% and 95%, respectively.^7 32 64^

In non-fatal cases, the fever subsides and the patient’s condition improves over 1–3 weeks, although renal damage^65^ along with neurological effects and fatigue^66^ can persist for several weeks. Deafness is a common side effect during the convalescent phase, typically accompanied by neurological dysfunction and vertigo.^27 34 67^ In half of these cases, hearing returns partially after 1–3 months.

### Molecular epidemiology

Members of the arenavirus family are composed of an ambisense RNA genome and a nucleoprotein (NP), surrounded by a lipid envelope and a glycoprotein. The LASV genome consists of two single-stranded RNA segments: the small segment (S, 3.5 kb) encodes the NP and the glycoprotein precursor (GPC); the large segment (L, 7.2 kb) encodes the RNA-dependent RNA polymerase (LP) and the matrix protein (Z).

**Figure 2:**
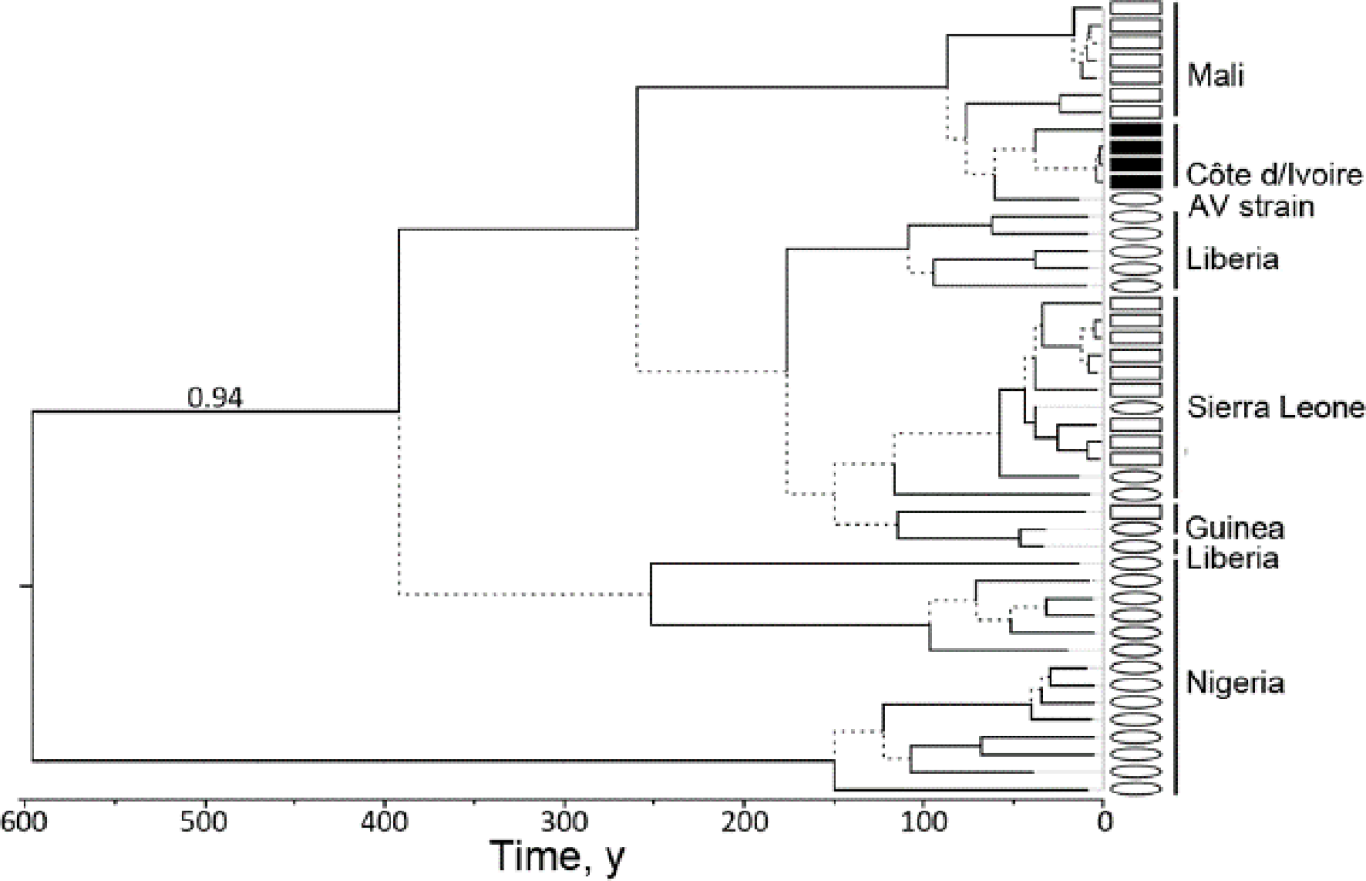
Bayesian chronogram of LASV based on the genomic L segment. LASV sequences of human origin indicated by ovals and sequences of *Mastomys natalensis* indicated by squares (black squares indicate ‘strain AV’ sequences). This tree was built under the assumption of a molecular clock and is therefore rooted. Source: Kouadio *et al.* 151 LASV, Lassa fever virus.

Once thought to be genetically stable, considerable genomic variation has been detected for geographically distant LASV strains, with phenotypic differences even among closely related isolates. LASV phylogeny is far more genetically diverse than Ebola virus in that <75% sequence is conserved for LASV compared with >97% for Ebola.^68 69^ LASV comprises at least four lineages: the prototype LASV strain isolated from Eastern Nigeria is lineage I; strains isolated from Southern Central and Northern Central Nigeria are lineage II and III and the large group from Guinea, Liberia and Sierra Leone are lineage IV (with three distinct subclades including the Josiah strain). A fifth ‘strain AV’ lineage from Mali/Cote D’Ivoire has been proposed (figure 2)^16 70 71^ as well as a newly designated lineage VI strain linked to Togo.^72^

### Therapeutic efforts

The recommended course for clinical management of LASV is general supportive care with management of symptoms. There is no approved antiviral treatment; however, evidence indicates that Lassa responds to ribavirin if administered at early onset of symptoms. Oral ribavirin has been used for postexposure prophylaxis for persons at high risk of secondary infection.^15 41 42 48^ While ribavirin studies have demonstrated a noticeable decrease in mortality for severe cases, high fatality rates have been observed for both ribavirin-treated and untreated patients, which underscores the need for better Lassa therapeutics.^7 73^

Favipiravir is another broad-spectrum RNA inhibitor (licensed for influenza) that has broad-spectrum activity against RNA viruses and has been shown to decrease Lassa viremia in animal models.^74–78^ Small molecules with benzimidazole-related scaffolds possess activity against a variety of arenaviruses and can interfere with LASV processing or viral entry. One such compound (ST-193) has been assessed in an animal model of Lassa fever, where Lassa-treated guinea pigs exhibited fewer signs of disease and enhanced survival.^79 80^ A similar optimised analogue (ST-161) had subnanomolar activity against LASV using a plaque reduction assay with live virus.^81^

Small interfering RNA particles (siRNA) can been shown to inhibit LASV replication system for *in vitro* studies.^82^ NP-directed and L-directed siRNAs demonstrated antiviral activity in a Lassa vero cell assay against five Lassa isolates. Both siRNAs inhibited replication of virus strains by up to 1 log unit with no apparent effect on cell viability. However, siRNA candidates must perfectly base pair to the target sequence, which could be a significant roadblock given the amount of genomic variation with LASV.

Monoclonal antibodies specific for LASV neutralisation cloned from West African Lassa fever survivors^83^ appear to bind to individual or combined Lassa GP protein subunits, which can potently neutralise all four LASV lineages—an early start to immunotherapeutic development and vaccine design.^84^ In particular, in the recent elucidation of the crystal structure of the trimeric, prefusion ectodomain of LASV GP bound to human neutralising antibody may accelerate development of antibody-based LASV therapeutics.^85^

### Vaccine efforts

Currently, there is no vaccine that protects against LASV, and there are no vaccines available for use in animals to reduce zoonotic transmission.^1 2^ To stimulate vaccine development, WHO finalised a Lassa fever vaccine Target Product Profile (TPP) in June 2017, which was made available to target vaccine scientists, product developers, manufacturers and funding agencies after public consultation.^10^ In response, LASV was made one of the priorities for vaccine development funding by the multiagency Coalition for Epidemic Preparedness Innovations (CEPI), using a multisector partnership approach to finance and develop vaccines for infections of epidemic potential.^86 87^

A number of different platform technologies have been evaluated as potential Lassa fever vaccines, including virus-like particles,^88 89^ DNA vaccines,^90 91^ non-replicating (inactivated) viruses such as Lassa^92^ and Yellow Fever (17D),^93 94^ Chimpanzee adenoviral vectors,^95^ recombinant Vesicular stomatitis virus (VSV/LASV)^96–98^ and reassortment viral vectors such MOPV/LASV.^99–101^ For the vaccine platforms, recombinant VSV/LASV and reassortant ML29 are considered the most advanced vaccine candidates in clinical development.^102 103^ Despite promising preclinical evaluation, none of these vaccine candidates have yet advanced to human clinical trials.

## Lassa diagnostics

Laboratory diagnosis of LASV infection is made by detection of the virus (culture), LASV RNA, LASV-specific IgG or IgM antibody response or LASV antigens shed during replication. LASV RNA is detected using a nucleic acid amplification test, which can include techniques such as PCR, loop-mediated isothermal amplification (LAMP) and strand displacement assays. Antibodies and antigens can be detected by indirect immunofluorescence assay test (IFA or IIFT), western blot (WB), ELISA or rapid diagnostic test (RDT) formats. Active infections can be diagnosed by virus isolation, PCR, LASV antigen positivity or IgM, along with clinical symptoms consistent with Lassa fever.

Diagnostic test types differ in complexity, infrastructure requirements and appropriateness for a rapid response. An overview of the implementation requirements for the range of diagnostics available for LASV is shown in table 1.

**Table 1:** Diagnostics infrastructure comparison

Test type	Lab infrastructure requirements (example)	Training requirements (example)	Turnaround time	Typical cost	In-house test	Commercial source
Virus isolation, neutralisation	HIGH (BSL-3/4) (reference laboratory)	HIGH (advanced lab technician)	7–10 days	–	>3	–
NAAT reference (includes multiplex)	HIGH (BSL-3/4) (reference laboratory)	HIGH to MODERATE (advanced lab technician)	2–3 hours 1–2 hours (prep)	$30–$100	>10	3–5
NAAT POC	MODERATE/BSL-2 (district hospital)	MODERATE (lab technician)	1–2 hours	$15–$30	–	–
ELISA, IFA/IIFT, WB	HIGH to MODERATE (regional lab, district hospital)	MODERATE (lab technician)	3–4 hours	$5–$15	>10	1–2
RDT	LOW (clinic, health centre, field settings)	LOW (nurse, healthcare worker)	<30 min	$1–$20	–	1

IFA/IIFT, indirect immunofluorescence assay test; NAAT, nucleic acid amplification test; POC, point of care; RDT, rapid diagnostic test; WB, western blot.

The majority of international laboratories use in-house LASV assays (see tables 2 and 3), with reports suggesting an equal number of published and unpublished protocols.^104^ Commercial assays for PCR and serology are available but are primarily labelled for research use only (see online supplementary tables S1 and S2).

### Lineage detection

Lassa lineage diversity must be considered in the choice of PCR primers and serology antigens, as test sensitivity relies on homology between the unknown specimen and the molecular probes or proteins used for detection. As more lineages and clades are revealed, and as mutations occur, tests will need to be revalidated and enriched to encompass variations observed at the nucleotide (20%–25%) and amino acid (8%–12%) levels.^66^ For some use cases, tests developed for a specific lineage or region may be appropriate; other cases may require detection of all known strains.^62 105–108^

### Specimen diversity

LASV can be found in many body fluid compartments during infection besides blood, including urine,^19 24 109 110^ semen,^11 32 44 45^ cerebrospinal fluid,^66 111^ with evidence from throat swabs.^41 111^ There is evidence that acute infections detected in the CNS can report as negative in blood.^112^ LASV can persist in the central nervous system, urine and semen long after viral clearance in the blood, possibly in immunologically protected compartments.^66 113 114^

Viral titre is highly predictive of disease outcome.^115^ LASV RNA was detected as early as 3 days after infection in monkeys, and viral load peaked between 6 and 12 days in survivors but increased until death in fatally infected animals.^116^ For patients ill enough to be admitted to hospital, the average viral load in serum is 10^3^–10^4^ copies/mL, which clears within 21 days for survivors.^66 109 117 118^ Patients with viral load>10^8^ copies/mL typically progress to multiorgan failure and shock, with viremia increasing to 10^8^–10^9^ copies/mL days before death;^119^ similar levels were found for cultured cell titres >10^3^ TCID_50_/mL.^111 120^

Fatality has also been linked to suppressed or diminished IgM response throughout infection, supporting the hypothesis that an early and vigorous humoral response is critical to surviving infection.^114 121^ In monkeys, IgG response can appear as late as 12 days postinfection for both fatal and surviving cases, but titres were much higher in the survivors.^116^

### Molecular diagnostics

Molecular diagnostic tests (PCR, LAMP and similar assays) are designed to detect highly conserved regions of the pathogen genome and are commonly used as the most sensitive method to detect active infection. Molecular diagnostic tests for LASV generally target the S genome segment encoding the GPC or the NP regions (table 2). Tests targeting the LP genome segment are non-specific for Old World arenavirus detection. Given the high mutation rate and genetic diversity of LASV, molecular diagnostic tests must encompass this sequence diversity or otherwise risk false negatives in mutated or ‘uncovered’ strains. Amplicons produced by the older tests can be sequenced to evaluate homology to known strains and new probes can be generated from sequencing data to improve lineage sensitivity.^19 122 123^ In some cases, multiple PCR assays could be performed to enhance coverage (see online supplementary table S1).

10.1136/bmjgh-2018-001116.supp2 Supplementary data

**Table 2:** In-house NAAT tests for LASV

Assay type	Target	Multiplex	Detection	Detection limit	Reference
RT-PCR	S segment (GPC)	–	Acrylamide gel	10^2^ TCID_50_	110
RT-PCR	S segment (GPC)	–	Agarose gel/ Southern blot	10^1.6^ TCID_50_	118
RT-PCR	S segment (GPC)	–	Standard	1–10 copies/assay	45 109 144
real-time RT-PCR (qRT-PCR)	S segment (GPC)	–	SYBR-green	8.6–16 copies/assay 1545–2835 copies/mL	45 144 152
RT-PCR	S segment (NP)	10-plex	Mass spectrometry	20 copies/assay	138
RT-PCR	S segment (NP)	>20-plex	Microarray	1900 copies/assay	139
RT-PCR	L segment (LP)	–	Standard	4290 copies/mL	144 153
RT-PCR	L segment (LP)	8-plex	Standard	1200 copies/assay 10^5^ copies/mL	137
RT-PCR	S segment (GPC)	–	Standard	4–30 copies/assay 342–2560 copies/mL	45 107 144
qRT-PCR	S segment (GPC), L segment (LP),	–	SYBR-green	n/a	45 123
One-step qRT-PCR	S segment (GPC, NP)	–	Standard	234–583 copies	45 141
RT-PCR	S segment (GPC), L segment (LP)	–	Standard	4290 copies/mL (serial assays)	122 144
RT-LAMP	S segment (GPC)	–	Turbidity	100 copies	154
One-step RT-PCR	S segment (GPC)	–	Microarray	1540 copies/mL	45 155
One-step qRT-PCR	S segment (GPC)	4-plex	Standard	45–150 copies/assay	136
One-step RT-PCR/LDR	L segment (LP)	11-plex	Microarray	100 copies/mL	140

LAMP, loop-mediated isothermal amplification; LASV, Lassa fever virus; LDR, ligase detection reaction; NAAT, nucleic acid amplification test; RT, reverse transcriptase; q, quantitative.

### Serological and antigen detection assays

Serological tests can be used to detect IgM and IgG antibodies raised against LASV antigens as well as direct capture and detection of LASV antigens. LASV NP, GP and Z proteins have been shown to be immunogenic.^124 125^ IgM and antigen tests can be used to detect active infection, although not all patients have detectable IgM at the acute stage^113 126^ and both IgM and IgG antibodies can be immunosuppressed in severe cases.^113 127 128^ Particularly for endemic settings, IgG is generally used only for surveillance.^11 129 130^

LASV antigen detection has been demonstrated to be a robust method for detection of active infection. While genetic sequence diversity and minor genetic mutations typically have less of an impact on protein sequence, early antigen tests have shown some variation in LASV lineage sensitivity. In tandem, detection of LASV NP antigen and anti-LASV IgM demonstrated 88% sensitivity and 90% specificity for early stage infection, sufficient for diagnosis in ~90% of PCR-positive cases.^108 131^

Serology test platforms for LASV include IFA/IIFT, ELISA, WB, multiplex bead assays and one RDT (table 3). IFA tests using infected Vero cells were the traditional method for LASV serodiagnosis, but have been largely displaced by ELISA due to time and biosafety constraints.^45^ Most serological tests were developed using antigens from LASV lineage IV (Josiah strain), though the AV strain has also been used.^132 133^ A small number of ELISA kits are commercially available, although primarily marketed as research-use only (see online supplementary table S2).

10.1136/bmjgh-2018-001116.supp3 Supplementary data

### Rapid tests

LASV RDTs, which leverage the same antibody/antigen capture agents as an ELISA but packaged in a stripped-down lateral flow format, can play an important role for patient care and outbreak response in outlying laboratories and clinics. A dipstick-based RDT for LASV has been developed that detects NP from fingerstick whole blood specimens.^7 108 134 135^ For LASV lineage IV (Josiah strain), the dipstick LASV RDT performed with good sensitivity (91% sensitivity, 86% specificity) compared with its progenitor ELISA (94% sensitivity, 84% specificity; both relative to qPCR). The monoclonal capture agents developed for this LASV RDT showed reduced sensitivity to LASV lineages II and III; improvements to the assay using a polyclonal approach suggest increased pan-lineage antigen sensitivity.^108^

**Table 3:** In-house serological and antigen tests for LASV

Assay type	Target	Lineage	Multiplex	Reference
ELISA	IgG, IgM, Ag	Lineage IV	–	128
Immunoblot	IgG, IgM	Lineage IV (NP)	–	38
ELISA	IgM, NP Ag	Lineage IV	–	41 131
ELISA	IgG, IgM	Lineage IV	–	129
ELISA	IgG, NP Ag	Strain AV (NP)	–	133
ELISA	IgG, IgM	Lineage III, IV, V	–	106
ELISA	NP Ag	Lineage IV	–	7
ELISA	IgG, IgM	Strain AV (NP)	–	132
RDT	NP Ag	Lineage IV	–	7 45
RDT	NP Ag	Lineage II, III, IV	–	108
Bead assay	IgG	Lineage IV (GP, NP)	7-plex	45 143
Bead assay	IgM, NP, GP	Lineage IV	LASV/EBOV	45 142

EBOV, Ebola virus; LASV, Lassa fever virus; RDT, rapid diagnostic test.

### Syndromic approach

At the early stages, the symptoms of Lassa fever can mimic other endemic diseases such as malaria, typhoid fever and other VHFs. A syndromic approach that tests for pathogens based on a syndrome such as VHF, using multiplex panels to quickly identify or eliminate likely pathogens from a single specimen, could be more effective in expediting LASV outbreak detection.

Several groups have demonstrated multiplex PCR assays for differential detection of VHF including Lassa. Real-time RT-PCR assays were developed for 28 VHFs to be processed as 4-plex reactions^136^ and 8-plex reactions.^137^ To overcome the limitations of fluorescence detection, combining RT-PCR with tagged primers can enable detection of 64 distinct species using mass spectrometry.^138^ Others have paired conventional assays with multiplex detection formats such as bead-based platforms^139 140^ and microarrays.^140^ A comprehensive set of 48 TaqMan-based PCR assays has been developed that can enable large-scale parallel processing of VHF agents.^141^ In addition, PCR panels for febrile agent and biothreat panels (5, 20 and 26-member panels including Lassa) are commercially available in bead-based and real-time TaqMan format (see online supplementary table S1).

A multiplex bead-based immunoassay platform for differential diagnosis using antigens and IgM for Lassa and Ebola demonstrated greater detection sensitivity for LASV GP antigen and IgM (25× and 5×, respectively) than conventional ELISA.^142^ A similar approach was used to survey pathogen exposure in West Africa for multiplex IgG detection of Lassa, Ebola, Marburg, Rift Valley fever and Crimean-Congo Haemorrhagic Fever as well as pan-flavivirus and pan-alphavirus.^143^

## Challenges for Lassa diagnostics

As laboratory-developed protocols are generally not manufactured for distribution or regulated by an international agency, there remains a need for commercial pan-LASV tests with assured and reproducible quality that can be procured by any international laboratory or agency. The ideal LASV diagnostic test would detect all known LASV lineages with high sensitivity. While PCR enables the most sensitive platform for early detection, the high genetic diversity of LASV may result in false negatives for older tests, and to date, only a few attempts at pan-LASV PCR probe sets have been demonstrated. And while antigen tests are typically less dependent on minor genetic mutations, early results have shown some variation in lineage sensitivity, with further work underway to demonstrate pan-Lassa validation.^108^

Validation and quality assessment can be valuable in evaluating the entire testing process, including sample preparation, method of amplification or capture, detection methods and lineage sensitivity. Even for identical protocols, laboratory proficiency can significantly impact the quality of results. In 2004 and 2015, external quality assessments (EQAs) were conducted by the European Network for Diagnostics of Imported Viral Diseases using well-characterised Lassa panels for 24 international laboratories.^104 144^ Early LASV detection rates varied from 50% to 85.7%, with later improvement of 58% to 100%; however, 7 of 24 labs fell below good-to-acceptable performance and 11 labs reported false-negative results.

Sourcing specimens for clinical validation can be a major roadblock for both diagnostic development and quality control. International reference standards and proficiency panels could assist development and validation of diagnostic tests and also help improve laboratory proficiency and EQA efforts towards quality. Several international reference institutes, including the WHO International Biological Reference custodian laboratories, could be sources for specimens for development and validation (see online supplementary list S1). As new diagnostic tests are validated, these agencies could assist in routine EQA monitoring of test performance using up-to-date clinical specimen panels and reference standards.

10.1136/bmjgh-2018-001116.supp1 Supplementary data

## Conclusion

Diagnostics are essential for the recognition and control of outbreaks of LASV, one of the most widespread and genetically diverse agents of VHF. Improved LASV tests are needed for endemic clinical management, outbreak response and vaccine and therapeutic clinical trials. Surveillance across all LASV lineages and regions will continue to be important to rapidly identify ‘hot spots’ for intervention and containment, and to monitor genetic and geographical shifts in both human and animal reservoir populations. For vaccine development, diagnostics are fundamental to detecting and differentiating an infection challenge from vaccine immunity. Finally, there is strong consensus that early diagnosis and treatment increases the survival rate from LASV infection.

This review has identified test resources for Lassa molecular diagnostics and serology and further described a large number of in-house LASV tests used across the international community. However, several gaps identified in the 2016 WHO R&D Blueprint remain.^8 145^ Efforts should be made to advance existing diagnostic platforms towards clinical validation and regulatory approval. Implementation of diagnostics could be further refined with more detailed understanding of LASV kinetics across a range of sample types, and a more robust point-of-care or field-appropriate design where appropriate. Tests that are currently available could benefit from EQA with the goal of standardisation of test sensitivity, specificity and lineage/regional coverage.

Given the challenges identified for LASV detection in endemic and often low-resourced settings, rapid on-site diagnosis of suspect cases may bridge the gap. A range of assays have been already developed for commercial point-of-care platforms, thus it is possible that current LASV assays could be adapted into a cartridge-based format. The combination of rapid, point-of-care antigen detection with a point-of-care confirmatory test has been suggested as an ideal implementation for early case detection and outbreak response.^108 146 147^

Each of the aforementioned diagnostic use scenarios have different performance requirements for text complexity, sensitivity, specificity and turnaround time. Development of new and improved LASV diagnostics could be facilitated by a clearly defined set of use cases to describe where and how diagnostic tests are most needed, coupled with TPPs with detailed performance characteristics for the highest priority molecular and immunodiagnostics.^148^ While numerous barriers to achieving regulatory approval exist,^142^ initiatives from WHO and other organisations such as the Foundation for Innovative New Diagnostics (FIND) and CEPI are intended to make development and commercialisation of LASV diagnostics more feasible.^86 148–150^
