# Supplementary material for: Diagnostics for Lassa fever virus: a genetically diverse pathogen found in low-resource settings
Source: BMJ Glob Health. 2019 Feb 7;4(Suppl 2):e001116. doi: 10.1136/bmjgh-2018-001116 (PMC6407561; doi:10.1136/bmjgh-2018-001116)
Supplement: Supplementary data [file bmjgh-2018-001116supp002.pdf]

Table S1: Molecular Diagnostic Tests for LASV

Commercial and regulated assays for Lassa are presented. Legacy lab-developed tests and in-house assays are not presented here, as sensitivity/specificity/LOD data is lab-specific.

Developer	System	Regulation	Sample Type	Target	LOD
**Commercial – single assays**					
**Altona Diagnostics** GmbH (GER)	RealStar® Lassa Virus RT-PCR Kit 1.0	CE-IVD	not specified	Lassa virus (LASV) specific RNA.	qualitative, not specified
**Altona Diagnostics** GmbH (GER)	RealStar® Lassa Virus RT-PCR Kit 2.0	RUO	not specified	Lassa virus (LASV) specific RNA.	qualitative, not specified
**genesig** (UK) (Primerdesign Ltd)	Primerdesign™ genesig® Kit for Lassa virus Josiah (LASV-Josiah)	RUO	not specified	Lassa virus (LASV) specific RNA, LASV-Josiah genome	100 copies of target template
**LifeRiver** (China) Shanghai ZJ Bio-Tech Co., Ltd	Lassa Virus (LV)Real Time RT-PCR Kit	CE-IVD	Serum, non-heparin anticoagulant, feces, urine or body fluid sample	LV RNA	1×10^3 copies/ml
**LIPSGene/VL Diagonstics** (GER)	Lassa Virus (LV) RT-PCR Kit	CE-IVD	not specified	not specified	not specified
**Commercial – multiplex assays**					
**Developer**	**System**	**Regulatory status**	**Sample Type**	**Target**	**LOD**
**GenArraytion Biothreat, Febrile kit panels** (TaqMan or Luminex xMAP)	MultiFLEX: Febrile Agent Panel Mega Febrile Panel BioThreat Panel	RUO	not specified	not specified	<10 copies per rxn
