# Supplementary material for: Diagnostics for Lassa fever virus: a genetically diverse pathogen found in low-resource settings
Source: BMJ Glob Health. 2019 Feb 7;4(Suppl 2):e001116. doi: 10.1136/bmjgh-2018-001116 (PMC6407561; doi:10.1136/bmjgh-2018-001116)
Supplement: Supplementary data [file bmjgh-2018-001116supp003.pdf]

Table S2: Serology Tests for LASV

Commercial and regulated assays for Lassa are presented. Legacy lab-developed tests and in-house assays are not presented here, as sensitivity/specificity/LOD data is lab-specific.

Commercial ELISA and IFT/IFA					
**Developer**	**System**	**Regulatory status**	**Sample Type**	**Target**	**LOD**
**Zalgen Labs / Corgenix** (USA)	**ReLASV® Pan-Lassa Antigen ELISA Test Kit**	RUO	Serum, EDTA plasma, or citrated plasma	LASV Lineages II, III, IV nucleoprotein (NP) antigen	not specified
**Zalgen Labs / Corgenix** (USA)	**ReLASV® Pan-Lassa IgG/IgM ELISA Test Kit (NP/GP)**	RUO	Serum, EDTA plasma, or citrated plasma	Human IgG/IgM to LASV Lineages II, III, IV nucleoprotein (NP) and glycoprotein (GP) antigens	not specified
**Creative Diagnostics** (USA)	**Anti-LASV GP polyclonal antibody**	RUO	not specified	not specified	not specified
**Bernhard Nocht Institute** (GER)	in development	not specified	not specified	not specified	not specified
**Abbexa** (UK)	in development	not specified	not specified	not specified	not specified
**Commercial RDT**					
**Developer**	**System**	**Regulatory status**	**Sample Type**	**Target**	**LOD**
**Zalgen Labs / Corgenix** (USA)	**ReLASV® Antigen Rapid Test**	RUO	Whole blood from finger stick, serum or plasma samples	LASV Lineage IV nucleoprotein (NP) antigen	not specified
**Zalgen Labs / Corgenix** (USA)	**ReLASV® Pan-Lassa Antigen Rapid Test**	RUO	Whole blood from finger stick, serum or plasma samples	LASV Lineages II, III, IV nucleoprotein (NP) antigen	not specified
