# Supplementary material for: Diagnostics for Lassa fever virus: a genetically diverse pathogen found in low-resource settings
Source: BMJ Glob Health. 2019 Feb 7;4(Suppl 2):e001116. doi: 10.1136/bmjgh-2018-001116 (PMC6407561; doi:10.1136/bmjgh-2018-001116)
Supplement: Supplementary data [file bmjgh-2018-001116supp001.pdf]

List S1: International References

Several international reference institutes provide specimens for validation or EQA/proficiency. These groups typically have a defined pathogen/disease focus with a corresponding archive of biological reference materials, and the supplies may be limited.

- The European Network for Diagnostics of Imported Viral Diseases (ENIVD) established an expert laboratory network (EVD-LabNet, www.evd-labnet.eu) to provide expert laboratory support for networking, external quality assessments and training of laboratories involved in (re)emerging viral diseases, including inactivated Lassa cell culture supernatant.
- The National Institute for Biological Standards and Control (NIBSC, www.nibsc.org) provides a range of biological reference materials and reagents, including influenza reagents, Quality Control Reagents Unit reagents and other CE-marked IVD reagents.
- The Paul Ehrlich Institute (PEI, www.pei.de) has developed reference materials for serological testing, nucleic acid amplification techniques, and immunoglobulin preparations; PEI is also a resource for international standards and reference panels.
- INSTAND e. V. (www.instand-ev.de) is an interdisciplinary, not-for-profit, scientific medical society, organising EQAs as part of external quality assurance for nearly all areas of laboratory diagnostics.
- The European Virus Archive (EVAg, www.european-virus-archive.com) is a non-profit organisation with expertise in virology dedicated to the characterization, conservation, production, and distribution of biological materials in the field of virology.
- ATCC (www.atcc.org) is the premier global biological materials resource and standards organization offering an extensive collection of products and services manufactured under ISO certification and accreditation. The collection includes Quantitative Synthetic Middle East respiratory syndrome coronavirus (Lassa) RNA.
- The European Directorate for the Quality of Medicines & HealthCare (EDQM, www.edqm.eu) supplies chemical and biological reference preparations for the tests and assays to be carried out in accordance with the official methods prescribed in the European Pharmacopoeia.
- Quality Control for Molecular Diagnostics (QCMD, www.qcmd.org) is an independent International External Quality Assessment (EQA) / Proficiency Testing (PT) organisation for molecular diagnostics, providing laboratories with samples designed to resemble clinically significant specimens to assess specific analytical assay characteristics.
